# Dual-Specificity Tyrosine Phosphorylation-Regulated Kinase 3 Expression and Its Correlation with Prognosis and Growth of Serous Ovarian Cancer: Correlation of DYRK3 with Ovarian Cancer Survival

**DOI:** 10.1155/2024/6683202

**Published:** 2024-03-18

**Authors:** Jia Sun, Yingzi Zhang, Aijie Li, Hao Yu

**Affiliations:** ^1^Department of Obstetrics and Gynecology, The Second Affiliated Hospital of Shandong First Medical University, Taian, Shandong 271000, China; ^2^Department of Hepatobiliary Surgery, The Affiliated Taian City Central Hospital of Qingdao University, Taian, Shandong 271000, China

## Abstract

**Background:**

Epithelial ovarian cancer, primarily serous ovarian cancer (SOC), stands as a predominant cause of cancer-related mortality among women globally, emphasizing the urgent need for comprehensive research into its molecular underpinnings. Within this context, the dual-specificity tyrosine phosphorylation-regulated kinase 3 (DYRK3) has emerged as a potential key player with implications for prognosis and tumor progression.

**Methods:**

This study conducted a meticulous retrospective analysis of 254 SOC cases from our medical center to unravel the prognostic significance of DYRK3. Survival analyses underscored DYRK3 as an independent adverse prognostic factor in SOC, with a hazard ratio of 2.60 (95% CI 1.67-4.07, *P* < 0.001). Experimental investigations involved DYRK3 knockdown in serous ovarian cancer cell lines (CAOV3 and OVCAR-3) through a shRNA strategy, revealing substantial decreases in cell growth and invasion capabilities. Bioinformatics analyses further hinted at DYRK3's involvement in modulating the tumor immune microenvironment. In vivo experiments with DYRK3-knockdown cell lines validated these findings, demonstrating a notable restriction in the growth of ovarian cancer xenografts.

**Results:**

Our findings collectively illuminate DYRK3 as a pivotal tumor-promoting oncogene in SOC. Beyond its adverse prognostic implications, DYRK3 knockdown exhibited promising therapeutic potential by impeding cancer progression and potentially influencing the tumor immune microenvironment.

**Conclusions:**

This study establishes a compelling foundation for further research into DYRK3's intricate role and therapeutic potential in ovarian cancer treatment. As we unravel the complexities surrounding DYRK3, our work not only contributes to the understanding of SOC pathogenesis but also unveils new prospects for targeted therapeutic interventions, holding promise for improved outcomes in ovarian cancer management.

## 1. Introduction

Ovarian cancer remains a formidable global health challenge, standing as a leading cause of cancer-related mortality in women, with over 60% of cases classified as serous ovarian cancer (SOC) [1]. The intricate nature of ovarian cancer, compounded by the often-asymptomatic early stages and late-stage detection, contributes to its significant impact on overall patient prognosis [2].

The dual-specificity tyrosine phosphorylation-regulated kinase 3 (DYRK3) is an intriguing molecule in controlling the phase transition of membraneless organelles and plays critical roles in cancer biology [3]. Although its role in various physiological processes has been recognized, its exact function seems different in different malignancies. For example, DYRK3 was reported to be significantly downregulated in hepatocellular carcinoma compared with normal controls [4]. In contrast, DYRK3 can promote aggressiveness of glioblastoma via multiple signaling pathways [5, 6]. Nevertheless, DYRK3's role in other malignancies, specifically in ovarian cancer is not well characterized. Preliminary findings hint at its involvement in several cancer-associated pathways, particularly in modulating cellular growth and immune responses, making it a molecule of great interest in oncology research.

This research endeavors to bridge the existing knowledge gap by comprehensively investigating the expression of DYRK3 in serous ovarian cancer patients. Through a retrospective analysis of a substantial cohort and experimental manipulations of serous ovarian cancer cell lines, we aim to elucidate the correlation between DYRK3 expression, prognosis, and the underlying mechanisms influencing tumor behavior to offer a comprehensive exploration of DYRK3's potential as a prognostic marker and therapeutic target in serous ovarian cancer. As we delve into this exploration, we anticipate shedding light on DYRK3's significance and paving the way for novel therapeutic avenues in the management of ovarian cancer.

## 2. Methods

### 2.1. Ethical Approval

Prior to commencing the study, ethical approval was obtained from the Taian City Central Hospital Ethics Committee, ensuring that all research activities adhered to the Institutional Animal Care and Use Committee (IACUC) ethical standards and guidelines.

### 2.2. Cell Culture and Transfection

CAOV3 and OVCAR-3 human serous ovarian cancer cell lines, acquired from the American Type Culture Collection (ATCC, Manassas, Virginia, USA), were cultured in Dulbecco's Modified Eagle's Medium (DMEM) supplemented with 10% fetal bovine serum (FBS), 100 U/mL penicillin, and 100 *μ*g/mL streptomycin. Cells were maintained in a humidified incubator at 37°C with 5% CO_2_. For transfection, shRNAs targeting DYRK3 or scramble control shRNA were utilized, following the manufacturer's instructions (Cat. sc-39010-SH; Santa Cruz Biotechnology). Mock control was also conducted to serve as an additional control, which was only treated with transfection reagents. Cells were then subjected to immunoblotting to confirm knockdown efficiency and quantify the expression levels of filamin B and SOX9.

### 2.3. Cell Metabolism Assay

Transfected cells were seeded into 96-well plates at a density of 5000 cells per well and allowed to grow for designated time periods. The MTT assay was performed according to the manufacturer's instructions. Absorbance of the formazan dye, proportional to the number of viable cells, was measured spectrophotometrically at 570 nm using a microplate reader. Cell viability was calculated by normalizing to the control group, and results were expressed as mean ± SD.

### 2.4. Western Blotting

To extract total protein from cells, we utilized RIPA buffer enriched with protease and phosphatase inhibitors. The protein concentration was determined using the BCA protein assay kit. Electrophoresis was employed to segregate equal amounts of protein (20 *μ*g) loaded onto 12% SDS-PAGE gels. Subsequently, a wet transfer method was utilized to transfer the isolated proteins onto either nitrocellulose or PVDF membranes. Following an hour of blocking with 3% BSA in TBST, the membranes were incubated overnight at 4°C with primary antibodies. After TBST washing, secondary antibodies, HRP-conjugated, were applied for an additional hour. Visualization of protein bands was achieved using an ECL substrate, and an imaging apparatus captured the images. To normalize protein expression levels, either *β*-actin or GAPDH was employed as an internal control. Quantification of bands was performed using ImageJ.

### 2.5. Matrigel-Transwell Assay

Cells suspended in serum-free DMEM were placed in the upper chamber of a Matrigel (BD Biosciences)-precoated Transwell insert with an 8 *μ*m pore size for the invasion test. Each insert were seeded with 50,000 cells. The bottom chamber was filled with 10% FBS-containing media. After 48 hours, invading cells were fixed and stained with DAPI. The count of invading cells was determined from five randomly selected visual fields.

### 2.6. Clinical Cohort and DYRK3 Expression Analysis

A retrospective analysis was conducted on a cohort of 254 serous ovarian cancer (SOC) cases from our medical center (cases survived less than one-month were exclude in this study). DYRK3 expression levels were assessed through immunohistochemistry staining, and patients were grouped into low and high DYRK3 expression based on the median staining score. Clinical characteristics, including age, laterality, lymph node status, International Federation of Gynecology and Obstetrics (FIGO) stage, and chemotherapy, are summarized in Table 1 and analyzed for correlations with DYRK3 expression.

### 2.7. Survival Analyses

Overall survival analyses were performed using univariate log-rank tests. Additionally, multivariate Cox regression analysis was employed to evaluate the independent prognostic significance of DYRK3 expression along with other clinical variables, including age, laterality, lymph node status, and FIGO stage.

### 2.8. In Vivo Xenograft Experiments

The impact of DYRK3 knockdown on ovarian cancer growth was assessed in vivo using xenograft experiments with CAOV3 and OVCAR-3 cells. Growth curves and excised xenograft images were generated to visually represent the effects of DYRK3 knockdown on tumor growth in the in vivo microenvironment.

### 2.9. Gene Set Enrichment Analysis (GSEA)

We used the KEGG database to select gene sets for our analysis. The preselected KEGG gene sets were then mapped onto this ranked list. GSEA calculates an enrichment score (ES) that reflects the degree to which a gene set is overrepresented at the extremes (top or bottom) of the entire ranked list of genes. The ES is a measure of the gene set's deviation from an expected, uniform distribution. To assess the statistical significance of the enrichment scores, a permutation test was performed. This test generates a null distribution for the ES by permuting the class labels, which helps in evaluating the significance of the actual ES observed. The nominal *P* value and false discovery rate (FDR) were calculated for each gene set, providing a measure of the confidence we can have in the results. Finally, gene sets with a nominal *P* value < 0.05 and FDR < 0.25 were considered significantly enriched, indicating that the pathways they represent are likely to be key players in the biological processes differentiating the conditions studied [7].

### 2.10. Immune Cell Infiltration Analysis

Utilizing the TCGA dataset, we conducted a correlation analysis between DYRK3 expression and immune cell infiltration in ovarian serous cancers. Spearman correlation tests were performed to identify associations, and representative correlations, such as the positive correlation with Tcm cells and negative correlation with Th2 cells, were visualized.

### 2.11. Statistical Analysis

Statistical analyses were carried out using appropriate tests, including two-sided chi-square tests for clinical characteristics, log-rank tests for survival analyses, and Spearman correlation tests for immune cell infiltration analysis. Multivariate Cox regression analysis was employed to assess independent prognostic factors. All statistical analyses were conducted using R package. Results were considered statistically significant at *P* < 0.05.

## 3. Results

### 3.1. Stratification of Clinical Characteristics Based on DYRK3 Expression Levels

**Figure 1:**
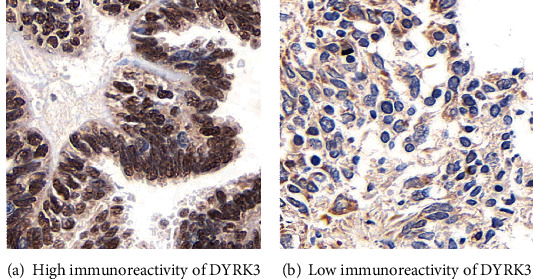
DYRK3 protein expression in ovarian cancer tissues. (a) Immunohistochemistry staining depicting representative high DYRK3 protein expression in ovarian cancer tissues. The immunoreactivity is prominently localized in the nucleus, with additional cytosol staining. (b) Representative low DYRK3 protein expression in ovarian cancer tissues. Magnification: 400x.

Table 1 provides a comprehensive overview of the clinical characteristics of the 254 serous ovarian cancer patients included in this study, stratified by DYRK3 expression levels. The cohort was divided into two groups based on DYRK3 median expression levels (Figure 1), a low-DYRK3 group (*n* = 127) and a high-DYRK3 group (*n* = 127).

In terms of age distribution, 153 patients were aged 60 years or younger, with 71 and 82 individuals in the low- and high-DYRK3 groups, respectively. Conversely, among the 101 patients aged over 60 years, 56 and 45 were categorized into the low- and high-DYRK3 groups, respectively (*P* = 0.158). The laterality of ovarian cancer manifestation revealed a trend, although not statistically significant, with 190 patients presenting unilateral tumors and 64 patients displaying bilateral tumors. Notably, lymph node status exhibited a significant association with DYRK3 expression levels (*P* = 0.010), indicating a correlation between DYRK3 and the presence of lymph node metastasis. Further, the FIGO stage demonstrated a pronounced correlation with DYRK3 expression levels (*P* < 0.001), with higher DYRK3 expression associated with advanced stages (stages III and IV). Interestingly, chemotherapy history did not show a significant correlation with DYRK3 expression levels (*P* = 0.257). These findings collectively suggest that DYRK3 expression levels may serve as a potential indicator of disease severity and progression in serous ovarian cancer.

### 3.2. Prognostic Significance of DYRK3 Expression for Overall Survival

**Figure 2:**
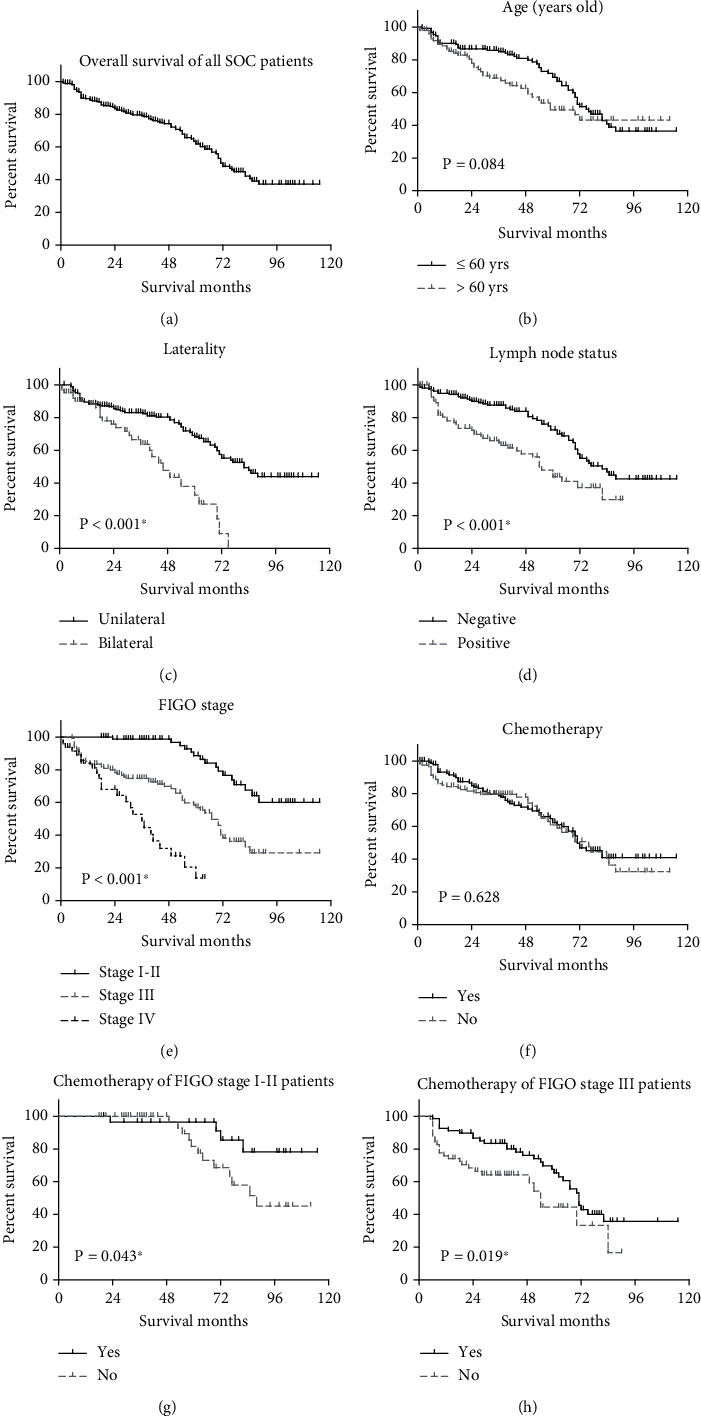
Overall survival analyses based on patients' characteristics. (a) The overall survival curves of all enrolled SOC patients or based on patients' (b) age, (c) laterality, (d) lymph node status, and (e) FIGO stage. The prognosis effect of chemotherapy treatment on the (f) entire cohort, (g) stage I-II patients, or (h) stage III patients was plotted, respectively. Data was plotted by Kaplan-Meier method and compared via log-rank test. ^∗^ indicates *P* < 0.05.

We next conducted univariate log-rank survival analyses to investigate the overall survival (OS) of 254 serous ovarian cancer patients (Table 2 and Figure 2). For the entire cohort, patients showed a median overall survival time of 72 months with a 5-year OS as 61.9% (Figure 2(a)). Stratification based on key clinical variables reveals compelling insights into the prognostic significance of these factors. For example, age, a fundamental demographic factor, exhibited a trend toward significance (*P* = 0.084, Figure 2(b)) in OS analyses. Patients aged 60 years or younger demonstrated a mean OS of 76.3 ± 3.5 months, with an associated 3-year OS rate of 85.0%. Conversely, patients over 60 years of age displayed a lower mean OS of 67.7 ± 5.0 months and a reduced 3-year OS rate of 68.8%. The laterality of ovarian cancer manifestation emerged as a highly significant determinant of survival outcomes (*P* < 0.001, Figure 2(c)). Patients with unilateral tumors exhibited a robust mean OS of 78.6 ± 3.3 months, corresponding to a 3-year OS rate of 83.1%. In contrast, those with bilateral tumors faced a substantially diminished mean OS of 44.8 ± 3.6 months and a 3-year OS rate of 63.6%.

Lymph node status, a critical indicator of disease spread, also significantly influenced OS outcomes (*P* < 0.001, Figure 2(d)). Patients with negative lymph node status displayed a prolonged mean OS of 80.6 ± 3.5 months, achieving a high 3-year OS rate of 87.6%. In contrast, those with positive lymph node status experienced a shortened mean OS of 53.4 ± 3.8 months, with a 3-year OS rate of 64.5%. Further, FIGO stage demonstrated a profound impact on OS (*P* < 0.001, Figure 2(e)), with increasing stage correlating with decreased survival. Patients in stages I-II exhibited a robust mean OS of 96.6 ± 3.7 months and an exceptional 3-year OS rate of 98.6%. Conversely, those in stage III and stage IV displayed diminishing mean OS values of 66.2 ± 4.2 months and 35.1 ± 3.5 months, respectively, accompanied by 3-year OS rates of 74.7% and 48.7%. Interestingly, although chemotherapy history did not show a significant impact on OS outcomes for the entire cohort (*P* = 0.628, Figure 2(f)), subgroup analyses indeed supported the survival advantages of chemotherapy administration. For example, in stage I-II patients, patients that accepted chemotherapy showed better OS than those that did not (*P* = 0.043, Figure 2(g)), and similar findings was observed in patients with stage III (*P* = 0.019, Figure 2(h)).

**Figure 3:**
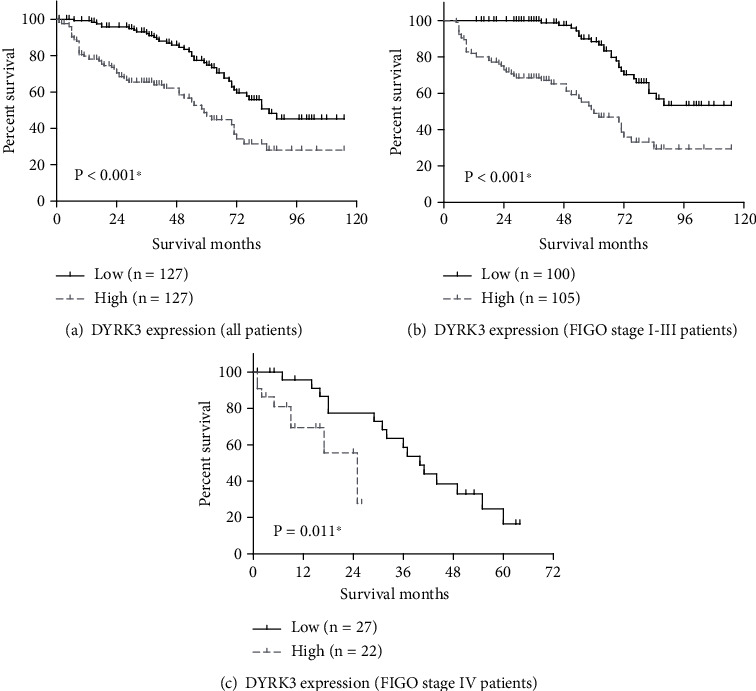
Overall survival analysis based on patients' DYRK3 levels. Patients were categorized into low-DYRK3 and high-DYRK3 groups based on the median staining score of DYRK3 expression. Kaplan-Meier survival curves depict the overall survival outcomes (a) in the entire cohort (*n* = 254), (b) in FIGO stage I-III patients (*n* = 205), and (c) in FIGO stage IV patients (*n* = 49). Data was plotted by Kaplan-Meier method and compared via log-rank test. ^∗^ indicates *P* < 0.05.

However, the most notable observation arises from DYRK3 expression levels, proving to be a highly significant predictor of survival (*P* < 0.001, Figure 3(a)). Patients with low DYRK3 expression exhibited a prolonged mean OS of 84.6 ± 3.6 months and an impressive 3-year OS rate of 92.1%. In contrast, those with high DYRK3 expression faced a significantly reduced mean OS of 60.2 ± 4.6 months and a 3-year OS rate of 65.4%. Additionally, the prognostic significance of DYRK3 was further confirmed in subgroup analyses regarding whether FIGO stage I-III patients (*P* < 0.001, Figure 3(b)) or stage IV patients (*P* = 0.011, Figure 3(c)).

The above findings underscore the potential of DYRK3 as a robust prognostic marker for overall survival in serous ovarian cancer; thus, we further outlines the results of multivariate Cox regression analysis, offering a nuanced perspective on the factors influencing survival SOC patients (Table 3). As a result, age emerged as a robust predictor of OS, with patients over 60 years facing a 2.21-fold increased hazard of mortality compared to their younger counterparts (95% CI 1.41-3.46, *P* = 0.001). The laterality of ovarian cancer, though exhibiting a trend (*P* = 0.050), did not reach statistical significance in the multivariate model. However, lymph node status, a critical indicator of disease spread, demonstrated no significant independent impact on OS (HR = 1.31, 95% CI 0.83-2.07, *P* = 0.244). Conversely, the FIGO stage proved to be a potent independent predictor of survival. Patients in stage III faced a 2.97-fold higher hazard of mortality compared to those in stages I-II (95% CI 1.55-5.67, *P* = 0.001), and patients in stage IV faced a substantially elevated hazard of 7.06 (95% CI 2.95-16.91, *P* < 0.001) compared to those in stages I-II. Importantly, DYRK3 expression levels emerged as a robust and independent predictor of OS in SOC patients. Patients with high DYRK3 expression had a hazard ratio of 2.60 (95% CI 1.67-4.07, *P* < 0.001), indicating a significantly elevated risk of mortality compared to those with low DYRK3 expression.

### 3.3. Cellular Effects of DYRK3 Knockdown in Ovarian Cancer Cells

**Figure 4:**
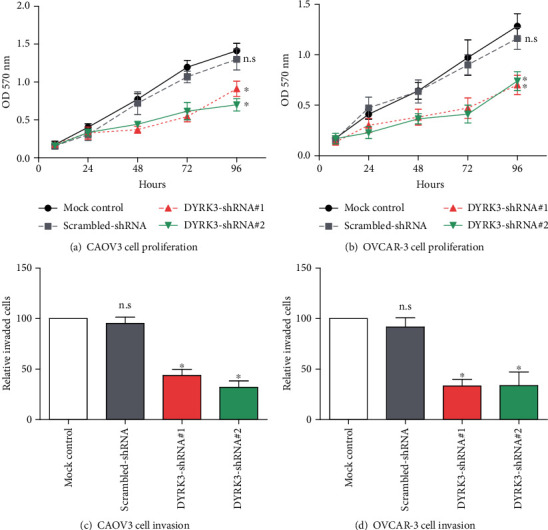
Cellular effects of DYRK3 knockdown in ovarian cancer cells. (a, b) MTT assay showing decreased cell growth in both CAOV3 and OVCAR-3 cell lines following DYRK3 knockdown by shRNAs. Control cells treated with scrambled-shRNA or mock control exhibit higher growth rates. (c, d) Matrigel-Transwell assay demonstrating impaired invasion capacities in both CAOV3 and OVCAR-3 cell lines upon DYRK3 knockdown. ^∗^ indicates *P* < 0.05.

DYRK3 knockdown using shRNAs demonstrated substantial cellular repercussions in ovarian cancer cells, compared to mock controls and scrambled-shRNA controls. The MTT assay revealed a significant reduction in cell growth for both CAOV3 and OVCAR-3 cell lines following DYRK3 knockdown (Figures 4(a) and 4(b)). Additionally, Matrigel-Transwell assays demonstrated impaired invasion capacities in both cell lines upon DYRK3 knockdown (Figures 4(c) and 4(d)), suggesting a potential regulatory role of DYRK3 in the invasive behavior of ovarian cancer cells. Next, we analyzed the correlation between DYRK3 with 1076 EMT-related genes in the TCGA dataset [8], which demonstrated that DYRK3 was positively and significantly correlated with 448 EMT-related genes, among which the top 10 were CD46, IRF6, MIR186, SMC5, HOOK1, ERMP1, PATJ, SBNO1, FLNB, and SOX9.

**Figure 5:**
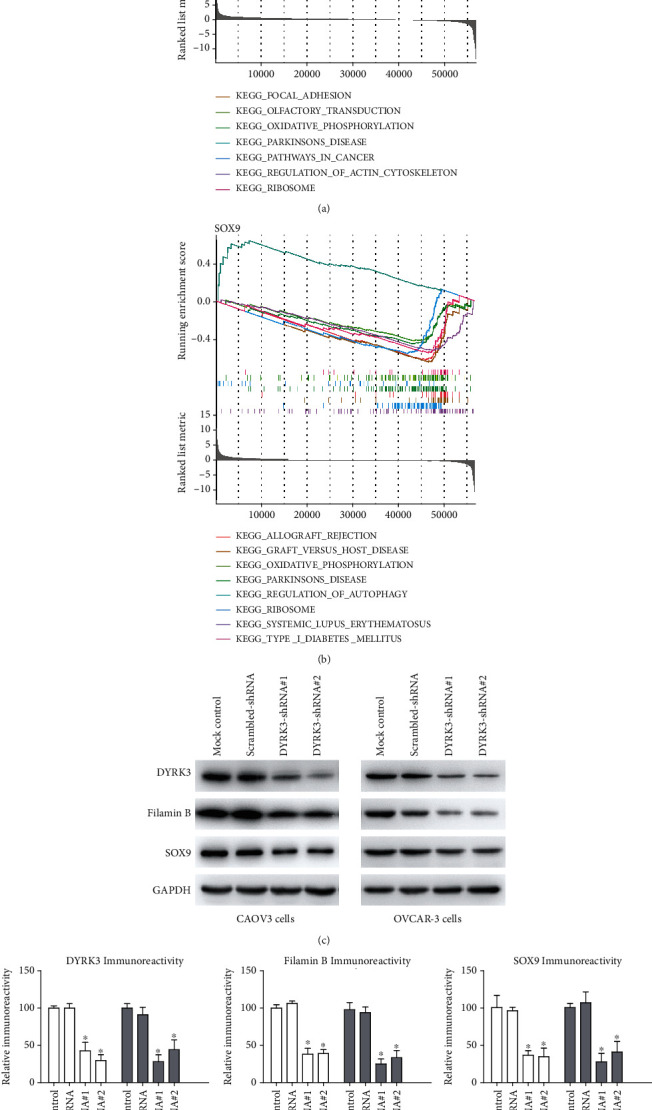
Pathway enrichment of FLNB and SOX9 in ovarian cancer: GSEA results. (a) Enrichment plots illustrating key pathways associated with FLNB in ovarian cancer, including ECM receptor interaction and oxidative phosphorylation. (b) Enrichment plots displaying significant pathways related to SOX9 in ovarian cancer, notably immune response pathways and oxidative phosphorylation. (c) Immunoblotting data confirming significant downregulation of filamin B and SOX9 protein levels following DYRK3 knockdown. (d) The immunoblotting data was semiquantified using ImageJ software and compared with the mock control group. ^∗^ indicates *P* < 0.05.

In ovarian cancer, the gene set enrichment analysis (GSEA) of FLNB and SOX9 genes revealed distinct and overlapping pathway involvements. FLNB is significantly associated with pathways like ECM receptor interaction, focal adhesion, olfactory transduction, oxidative phosphorylation, Parkinson's disease, pathways in cancer, regulation of actin cytoskeleton, and ribosome, indicating its broad impact on cellular processes crucial in cancer metastasis and fundamental cellular functions (Figure 5(a)). On the other hand, SOX9 showed enrichment in pathways related to immune responses such as allograft rejection, graft versus host disease, systemic lupus erythematosus, and type I diabetes mellitus, as well as oxidative phosphorylation, Parkinson's disease, regulation of autophagy, and ribosome, suggesting its role in modulating the immune environment in ovarian cancer (Figure 5(b)). The common pathways between FLNB and SOX9, like oxidative phosphorylation and ribosome, point to shared mechanisms affecting cellular metabolism and protein synthesis. Consistently, immunoblotting confirmed that DYRK3 knockdown significantly downregulated the protein levels of FLNB and SOX9 (Figures 5(c) and 5(d)). These findings highlight the diverse roles of FLNB and SOX9 in ovarian cancer, emphasizing their involvement in distinct and shared biological pathways with DYRK3.

### 3.4. Knockdown of DYRK3 Inhibits Ovarian Cancer Growth In Vivo

**Figure 6:**
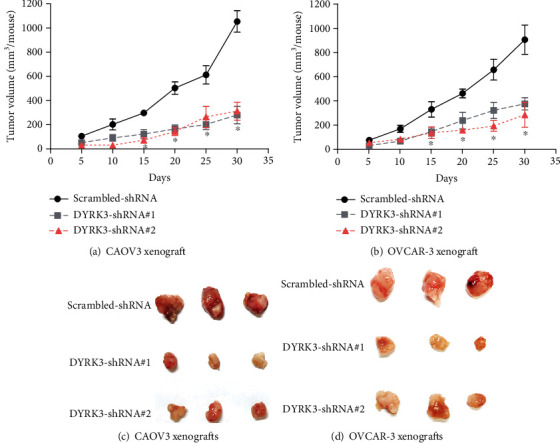
Knockdown of DYRK3 inhibits ovarian cancer growth in vivo. (a, b) Growth curves of xenografts established by CAOV3 and OVCAR-3 cells, demonstrating significantly inhibited tumor growth upon DYRK3 knockdown. (c, d) Excised xenograft images further validating the inhibitory impact of DYRK3 knockdown on ovarian cancer growth in vivo. ^∗^ indicates *P* < 0.05.

The in vivo impact of DYRK3 knockdown on ovarian cancer growth was assessed through subcutaneous xenograft experiments with CAOV3 and OVCAR-3 cells. Growth curves of established xenografts revealed a substantial inhibition in tumor growth upon DYRK3 silencing (Figures 6(a) and 6(b)). Consistent with these findings, the excised xenograft images (Figures 6(c) and 6(d)) visually validated the marked reduction in tumor size in the DYRK3-knockdown groups compared to controls. The in vivo data provides compelling evidence of DYRK3's pivotal role in promoting ovarian cancer growth in an in vivo microenvironment.

### 3.5. Correlation between DYRK3 Expression and Immune Cell Infiltration in Ovarian Serous Cancer

**Figure 7:**
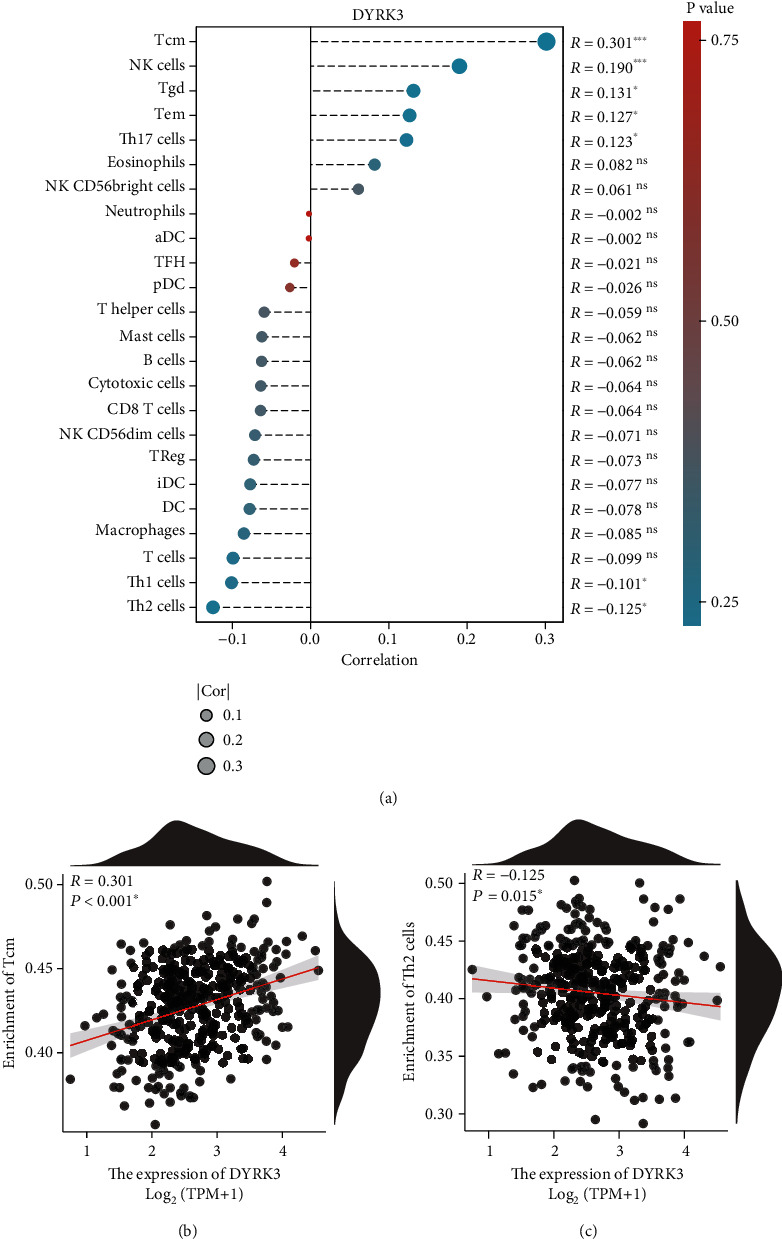
Correlation between DYRK3 expression and immune cell infiltration in ovarian serous cancer using the TCGA dataset. (a) Analysis of DYRK3 expression revealing correlations with immune cell infiltration in ovarian serous cancer. DYRK3 is positively correlated with the infiltration of Tcm cells, NK cells, Tgd cells, Tem cells, and Th17 cells while negatively correlated with Th1 cells and Th2 cells. Representative correlations include (b) a positive correlation with Tcm cell infiltration and (c) a negative correlation with Th2 cells.^∗^*P* < 0.05.

Utilizing the TCGA dataset, our analysis uncovered a compelling correlation between DYRK3 expression and immune cell infiltration in ovarian serous cancer. Examining 24 types of immune cells, DYRK3 displayed positive correlations with Tcm cells, NK cells, Tgd cells, Tem cells, and Th17 cells, while showing negative correlations with Th1 cell and Th2 cell infiltration (Figure 7(a)). Spearman correlation tests further validated these relationships, exemplified by the positive correlation with Tcm cell infiltration (Figure 7(b)) and the negative correlation with Th2 cells (Figure 7(c)). These findings shed light on the potential immunomodulatory role of DYRK3 in shaping the immune microenvironment of ovarian serous cancer. The intricate associations observed underscore the multifaceted impact of DYRK3 on immune cell populations, providing crucial insights for understanding the immune landscape in ovarian cancer.

## 4. Discussion

Our study delves into the multifaceted role of DYRK3 in serous ovarian cancer (SOC), providing novel insights into its potential as a prognostic marker and therapeutic target. The retrospective analysis of a sizable SOC cohort revealed that elevated DYRK3 expression is independently associated with adverse overall survival outcomes. Our findings align with emerging research suggesting DYRK3 as a crucial player in cancer biology [9]. While DYRK3 has been implicated in various malignancies, its specific role in ovarian cancer remained largely unexplored. Our study not only highlights DYRK3 as a tumor-promoting oncogene in SOC but also elucidates its potential as a therapeutic target by demonstrating the inhibitory effects of DYRK3 knockdown on cell viability, invasion, and in vivo tumor growth. By analyzing the correlation between DYRK3 and EMT-related genes [8] in TCGA-ovarian cancer dataset, we observed a positive correlation between DYRK3 with FLNB and SOX9, both of which had been well acknowledged as oncogenic molecules in tumorigenesis and cancer progression [10–13]. We also found that knockdown of DYRK3 can downregulate the protein expression of filamin B and SOX9. This points to DYRK3 being not just a prognostic marker but potentially a mechanistic driver of tumor progression [14]. The fact that age was also a significant factor affecting overall survival underscores the importance of early detection and targeted interventions [15].

The study also explored the interplay between DYRK3 expression and immune infiltration, a relatively less-explored dimension of this kinase. The positive correlation of DYRK3 with multiple immune cells like Tcm cells and NK cells, and its negative association with Th1 and Th2 cells, provides a hint into the complexity of the tumor microenvironment influenced by this kinase. Our data is consistent with a previous study focusing on the immune infiltration of colorectal cancers [16]. As reported by Laham et al., DYRK3 expression correlated with immune-infiltrating cells and was upregulated in MSI subtypes of colorectal cancers, highlighting their potential role as biomarkers for immunotherapy. It could be inferred that DYRK3 might be modulating the immune landscape, potentially leading to an environment more conducive to tumor growth and spread [17]. This observation aligns with the recent research that has started elucidating the broader roles of kinases in shaping immune responses.

Our study, however, is not without limitations. The retrospective nature of the cohort analysis may introduce biases, and the generalizability of our findings should be considered cautiously. Additionally, the precise molecular mechanisms through which DYRK3 modulates immune cell infiltration and influences cancer progression require further elucidation. Future research should focus on unraveling the intricate signaling pathways associated with DYRK3 in ovarian cancer and exploring its potential interactions with the tumor microenvironment.

## 5. Conclusions

Our study sheds light on the prognostic significance of DYRK3 in serous ovarian cancer and unveils its potential role in modulating immune cell infiltration. The inhibitory effects of DYRK3 knockdown on key cellular processes and in vivo tumor growth emphasize its promise as a therapeutic target. While our findings contribute to the growing body of knowledge on DYRK3 in cancer, ongoing research is essential to fully comprehend its intricate functions, paving the way for targeted therapies and personalized treatment approaches in ovarian cancer.

## Figures and Tables

**Table 1:** Clinical characteristics of SOC patients.

Variables	Cases (*n* = 254)	DYRK3 expression level		*P* value
		Low (*n* = 127)	High (*n* = 127)	
Age (years)				0.158
≤60 yrs	153	71	82	
>60 yrs	101	56	45	
Laterality				0.148
Unilateral	190	90	100	
Bilateral	64	37	27	
Lymph node status				0.010^∗^
Negative	156	88	68	
Positive	98	39	59	
FIGO stage				<0.001^∗^
Stage I-II	79	59	20	
Stage III	126	41	85	
Stage IV	49	27	22	
Chemotherapy				0.257
Yes	137	73	64	
No	117	54	63	

Abbreviations: LN: lymph node; SOC: serous ovarian cancer; DYRK3: dual-specificity tyrosine phosphorylation-regulated kinase 3; FIGO: International Federation of Gynecology and Obstetrics. Note: data was tested by two-sided chi-square test. ^∗^ indicates *P* < 0.05.

**Table 2:** Overall survival analyses of SOC patients.

Variables	Cases (*n* = 254)	OS (months)	3-year OS	*P* value
		Mean ± SD		
Age (years)				0.084
≤60 yrs	153	76.3 ± 3.5	85.0%	
>60 yrs	101	67.7 ± 5.0	68.8%	
Laterality				<0.001^∗^
Unilateral	190	78.6 ± 3.3	83.1%	
Bilateral	64	44.8 ± 3.6	63.6%	
Lymph node status				<0.001^∗^
Negative	156	80.6 ± 3.5	87.6%	
Positive	98	53.4 ± 3.8	64.5%	
FIGO stage				<0.001^∗^
Stage I-II	79	96.6 ± 3.7	98.6%	
Stage III	126	66.2 ± 4.2	74.7%	
Stage IV	49	35.1 ± 3.5	48.7%	
Chemotherapy				0.628
Yes	137	74.5 ± 3.9	78.8%	
No	117	70.2 ± 4.4	79.6%	
DYRK3 expression				<0.001^∗^
Low	127	84.6 ± 3.6	92.1%	
High	127	60.2 ± 4.6	65.4%	

Abbreviations: LN: lymph node; SOC: serous ovarian cancer; DYRK3: dual-specificity tyrosine phosphorylation-regulated kinase 3; FIGO: International Federation of Gynecology and Obstetrics. Note: data was tested by two-sided log-rank test. ^∗^ indicates *P* < 0.05.

**Table 3:** Multivariate analysis for overall survival of SOC patients.

Variables	HR	95% CI	*P* value
Age			
>60 vs. ≤60 yrs	2.21	1.41-3.46	0.001^∗^
Laterality			
Bilateral vs. unilateral	1.78	0.999-3.16	0.050
Lymph node status			
Positive vs. negative	1.31	0.83-2.07	0.244
FIGO stage			
Stage III vs. stage I-II	2.97	1.55-5.67	0.001^∗^
Stage IV vs. stage I-II	7.06	2.95-16.91	<0.001^∗^
DYRK3 expression			
High vs. low	2.60	1.67-4.07	<0.001^∗^

Abbreviations: HR: hazard ratio; 95% CI: 95% confidence interval; SOC: serous ovarian cancer; DYRK3: dual-specificity tyrosine phosphorylation-regulated kinase 3; FIGO: International Federation of Gynecology and Obstetrics. Note: data was analyzed by Cox hazard regression test. ^∗^ indicates *P* < 0.05.

## Data Availability

Original data will be available upon reasonable request.
